# Dr. Traill Green

**Published:** 1897-06

**Authors:** 


Dr. Traill Green died at Easton, Pa., April 29, 1897, aged 83 years. He was born at Easton, was graduated from the medical department of the University of Pennsylvania in 1835, and has practised medicine in his native city from that time until his retirement some years ago. In 1837 he was made professor of chemistry at Lafayette College, and in 1841 was called to the chair of natural sciences in Marshall College, Mercersburg. In 1866, Washington and Jefferson College conferred upon him the degree of LL. D. He built the astronomical observatory and presented it to Lafayette College and he also organised the Pardee scientific department at Lafayette. Dr. Green was, for nearly half a century, a member of the American Association for the Advancement of

Science. He was a member of many scientific societies, was the first president of the American Academy of Medicine, and an honorary Fellow of the American Association of Obstetricians and Gynecologists.

